# The Influence of Mitigation on Sage-Grouse Habitat Selection within an Energy Development Field

**DOI:** 10.1371/journal.pone.0121603

**Published:** 2015-04-02

**Authors:** Bradley C. Fedy, Christopher P. Kirol, Andrew L. Sutphin, Thomas L. Maechtle

**Affiliations:** 1 Department of Environment and Resource Studies, University of Waterloo, Waterloo, Ontario, Canada; 2 Big Horn Environmental Consultants, Sheridan, Wyoming, United States of America; Université de Sherbrooke, CANADA

## Abstract

Growing global energy demands ensure the continued growth of energy development. Energy development in wildlife areas can significantly impact wildlife populations. Efforts to mitigate development impacts to wildlife are on-going, but the effectiveness of such efforts is seldom monitored or assessed. Greater sage-grouse (*Centrocercus urophasianus*) are sensitive to energy development and likely serve as an effective umbrella species for other sagebrush-steppe obligate wildlife. We assessed the response of birds within an energy development area before and after the implementation of mitigation action. Additionally, we quantified changes in habitat distribution and abundance in pre- and post-mitigation landscapes. Sage-grouse avoidance of energy development at large spatial scales is well documented. We limited our research to directly within an energy development field in order to assess the influence of mitigation in close proximity to energy infrastructure. We used nest-location data (*n* = 488) within an energy development field to develop habitat selection models using logistic regression on data from 4 years of research prior to mitigation and for 4 years following the implementation of extensive mitigation efforts (e.g., decreased activity, buried powerlines). The post-mitigation habitat selection models indicated less avoidance of wells (well density β = 0.18 ± 0.08) than the pre-mitigation models (well density β = -0.09 ± 0.11). However, birds still avoided areas of high well density and nests were not found in areas with greater than 4 wells per km^2^ and the majority of nests (63%) were located in areas with ≤ 1 well per km^2^. Several other model coefficients differed between the two time periods and indicated stronger selection for sagebrush (pre-mitigation β = 0.30 ± 0.09; post-mitigation β = 0.82 ± 0.08) and less avoidance of rugged terrain (pre-mitigation β = -0.35 ± 0.12; post-mitigation β = -0.05 ± 0.09). Mitigation efforts implemented may be responsible for the measurable improvement in sage-grouse nesting habitats within the development area. However, we cannot reject alternative hypotheses concerning the influence of population density and intraspecific competition. Additionally, we were unable to assess the actual fitness consequences of mitigation or the source-sink dynamics of the habitats. We compared the pre-mitigation and post-mitigation models predicted as maps with habitats ranked from low to high relative probability of use (equal-area bins: 1 – 5). We found more improvement in habitat rank between the two time periods around mitigated wells compared to non-mitigated wells. Informed mitigation within energy development fields could help improve habitats within the field. We recommend that any mitigation effort include well-informed plans to monitor the effectiveness of the implemented mitigation actions that assess both habitat use and relevant fitness parameters.

## Introduction

Human-wildlife interactions are one of the primary issues in wildlife ecology and management. Human impacts to the environment are experienced globally [1] and estimates suggest that between one-third and one-half of earth’s land surface has been transformed by human action [2]. In order to maintain self-sustaining, healthy wildlife populations we need to minimize and mitigate the impacts of human endeavors. Mitigation of human development to benefit wildlife involves altering development practices to minimize or offset negative impacts to wildlife populations. Coincident with increasing human impacts are increasing energy demands. Energy development occurs in many prime wildlife habitats and can negatively affect wildlife populations [3,4]. Protection and development restrictions can help preserve wildlife populations; however, according to the World Database on Protected Areas (www.wdpa.org) only 13% of global terrestrial area is contained within protected-areas. Thus, globally, 87% of terrestrial lands are unprotected and candidates for potential development. Intelligently designed mitigation is the best option to support wildlife population persistence in areas were development will occur.

On-site mitigation involves a hierarchy of avoid, minimize, and restore [5,6]. Biodiversity offsets occur off-site and are an additional tool for “enhancing environmental value in situations where development is sought despite detrimental environmental impacts.” [7]. Biodiversity offsets and off-site mitigation are common and involve identifying areas outside of the development and focusing beneficial activities on those sites [5]. However, unless the off-site mitigation locations are protected, there are few guarantees those sites will not be developed. With current and future projected high rates of development in prime wildlife areas, it is conceivable that it will become more and more difficult to identify appropriate—un-impacted—off site locations for mitigation actions. Thus, mitigation within development areas is a critical tool to help reduce impacts to wildlife within development areas. Despite widespread energy development and mitigation suggestions, few studies have quantitatively assessed the efficacy of on-site mitigation efforts within energy development fields [8].

On-site mitigation suggestions within development areas typically involve techniques aimed at reducing human activity and disturbance. For example, within energy development areas in sagebrush steppe habitats, human use can result in both direct habitat loss and functional habitat loss as animals may avoid habitat in close proximity to infrastructure [9]. Habitat availability and quality is critical to species survival. Multiple studies have suggested mitigation techniques within energy development areas; however, very few studies have addressed the question of whether these suggested approaches are actually effective? To address this question, we examined the influence of on-site mitigation efforts on the distribution and abundance of important wildlife habitats within an energy development area.

Greater sage-grouse (*Centrocercus urophasianus*; hereafter sage-grouse) are an excellent species to assess the influence of on-site mitigation for a number of reasons. First, sage-grouse are a year-round resident species across sagebrush-steppe habitats in North America, and co-occur with many prime energy development areas. Second, sage-grouse responses to energy development are well documented and the species experiences several negative impacts within energy development areas [9–16]. Third, sage-grouse are likely an appropriate umbrella species for multiple other sagebrush obligate species [17,18]. Finally, sage-grouse are a conservation priority species and currently listed as “warranted, but precluded” from listing under the U. S. Endangered Species Act [19].

Accurate assessment of the influence of mitigation activities is possible after three conditions have been met. First, the impacts of unmitigated development have to be quantified and understood. Second, mitigation techniques need to be implemented on a relevant scale. Third, wildlife response to mitigation needs to be measured over a relevant time period. Sage-grouse are one of few species that have been studied long enough within energy development sites to meet these three criteria. The Powder River Basin (PRB) is located in Northeastern Wyoming and has been the site of extensive Coal Bed Natural Gas (CBNG) development since 2000. Research on the sage-grouse population in the region began in 2003. The research documented the impacts of oil and gas development in the region on sage-grouse from 2003 to 2007 [12,13] and provided further support for mitigation strategies recommended by other researchers. Recommended mitigation strategies included, burying power lines [20], abating road and well pad construction, and vehicle traffic [9,21]; and minimizing habitat for mosquitoes, the predominant vector for West Nile virus in sagebrush habitat [22], through management of water produced by active CBNG wells [12]. One of the major energy development companies in the region (Anadarko Petroleum Corporation [APC]) in cooperation with the Bureau of Land Management (BLM) implemented several of these mitigation strategies in 2008 in efforts to mitigate these wildlife impacts.

Sage-grouse are prey to multiple avian predators (e.g., eagles, hawks, ravens) and power lines and tall structures can provide perches for these predators. Additionally, roads and surface disturbance can result in both structural and functional habitat loss for sage-grouse [11]. Mitigation in the PRB designed to address these concerns involved reductions in road and well pad construction and total surface disturbance, and efforts to reduce traffic and noise [23] associated with energy development through remote well monitoring. Produced water from APC’s CBNG wells was transported by pipeline to treatment centers along the Powder River where it was then discharged into the Powder River instead of being stored in reservoirs. This action reduced the total surface area affected by development; limited mosquito habitat which could have increased exposure of sage-grouse to West Nile virus through infected mosquitos, and reduced subsidies for newcomer predators such as raccoons, (*Procyon lotor*), red fox (*Vulpes vulpes*) and striped skunk (*Mephitis mephitis*). It was impossible to isolate the effect of each unique mitigation action because the actions were implemented simultaneously within the development area. One of our objectives was to assess the cumulative effects of the mitigation actions on the abundance and distribution of important sage-grouse habitat within the energy development field. Essentially, we asked the question: did the mitigation efforts implemented produce measurable improvement in sage-grouse nesting habitats within the development area?

We addressed these questions using a before—after experimental design. We developed nesting habitat selection models for sage-grouse within the development area from 2004 to 2007. This was the pre-mitigation time frame. Therefore, the sage-grouse response to habitat structure characterized by the pre-mitigation habitat selection models represented sage-grouse responses to the pre-mitigated energy landscape. We also developed separate nesting habitat selection models for sage-grouse within the development area from 2008 to 2011. These nests represented the post-mitigation time frame. The sage-grouse response to habitat structure was characterized by the post-mitigation habitat selection models and represented sage-grouse responses to the post-mitigation energy landscape. Mitigation activities were targeted to reduce the impacts of energy infrastructure, including roads. Therefore, we predicted that successful mitigation would result in less avoidance of energy infrastructure and roads. Thus, application of pre- and post-mitigation nesting models to the same landscape should result in a greater abundance of high-quality nesting habitat and the post-mitigation models should predict higher quality habitats in closer proximity to energy infrastructure.

## Materials and Methods

### Study site

**Fig 1:**
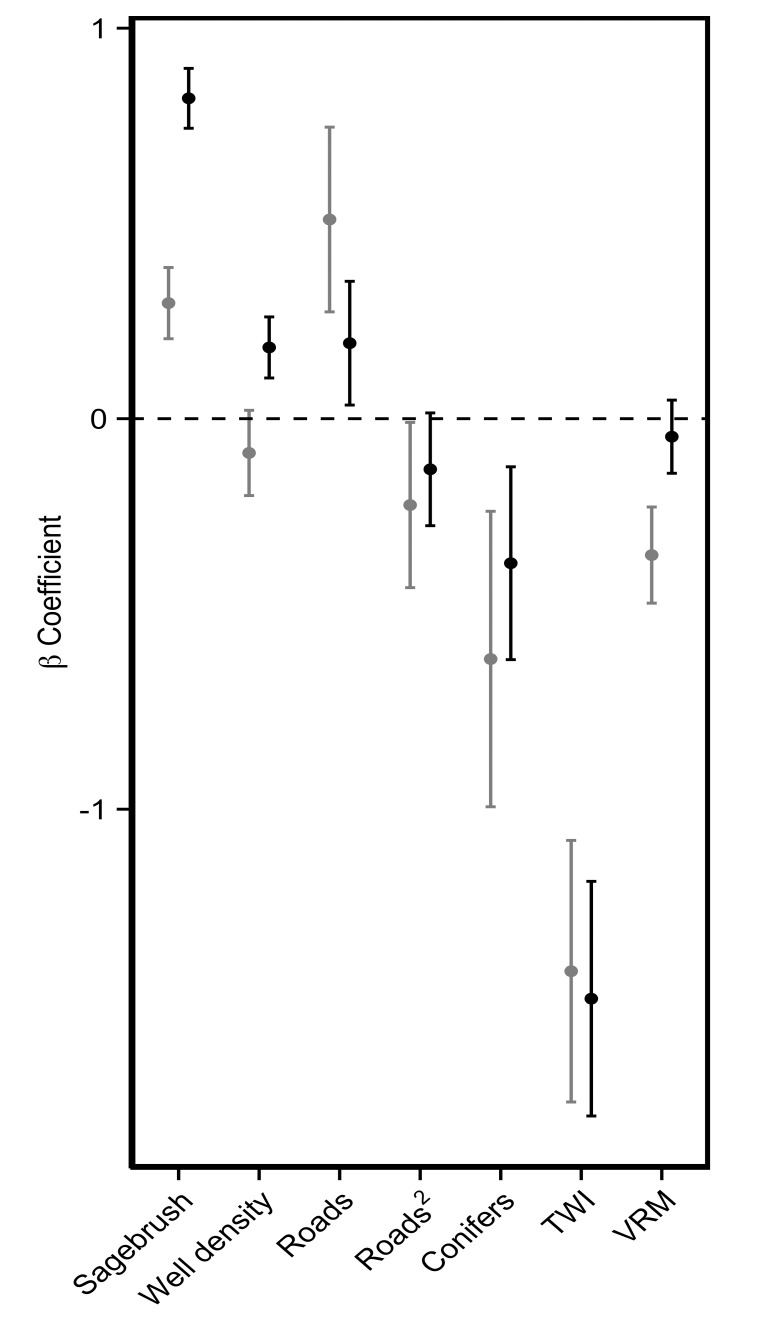
Coefficient Estimates. Standardized Beta-coefficients and associated standard errors for all variables included in the global Resource Selection Function models for greater sage-grouse nesting habitat models in the Powder River Basin, Wyoming, U.S.A. The gray lines represent estimates from the pre-mitigation model developed using nesting data from 2004–2007. The black lines represent coefficient estimates from the post-mitigation model developed using nesting data from 2008–2011. TWI: Mean Topographic Wetness Index. VRM: Mean Vector Roughness Measure.

Our study area was in the Powder River Basin and primarily in Johnson County with the northern portion extending slightly into Sheridan County, Wyoming, USA (106°20'2.538"W, 44°18'35.431"N; Fig 1). The study area encompassed 1612 km^2^ of which 65% was private, 29% was public land administered by the U.S. Bureau of Land Management (BLM), and 6% was Wyoming state land. Seventy four percent of the study area, including most of the private surface, contained federally owned mineral rights that were under the jurisdiction of the BLM. Cattle and sheep ranching were the primary agricultural uses and energy development, in the form of CBNG, was the primary energy extraction activity occurring in the study area. At the end of the study (2011) approximately 2548 CBNG wells were established in the study area. The study area was within the Great Plains Sage-Grouse Management Zone [19]. The study area includes part of the Powder River sage-grouse population [19] and provides year-round habitat for sage-grouse [13,24].

The climate in the study area was semi-arid and characterized by long cold winters and short summers. Monthly average temperatures ranged from 21.6°C in the summer to—5.8°C in the winter. Annual precipitation averaged 33 cm to 43 cm and average annual snowfall ranged from 84 cm to 170 cm. The majority of the study area was shrub-steppe habitat dominated by Wyoming big sagebrush (*Artemisia tridentata wyomingensis*). Plains silver sagebrush (*Artemisia cana cana*) was present but at much lower abundance and was largely limited to drainage corridors. The understory was composed of native and nonnative grasses and forbs including, western wheatgrass (*Pascopyrum smithii*), thickspike wheatgrass (*Elymus lanceolatus*), prairie junegrass (*Koeleria macrantha*), Sandberg bluegrass (*Poa secunda*), blue grama (*Bouteloua gracilis*), Japanese brome (*Bromus japonicus*), cheatgrass (*B*. *tectorum*), scarlet globemallow (*Sphaeralcea coccinea*), field sagewort (*Artemisia campestris*), curlycup gumweed (*Grindelia squarrosa*), milkvetch (*Astragalus bisulcatus*), and sweetclover (*Melilotus officinalis*). Conifers, primarily mountain juniper (*Juniperus scopulorum*), were not abundant in the study area and when present were concentrated along draws.

### Data collection

The before and after treatment components of the study were conducted by different research teams. However, every effort was made to maintain the exact methodological approaches after the mitigation to ensure comparable datasets. We summarize the methodological approaches briefly, focusing particularly on the post-treatment data collection. Details on pre-mitigation data collection have been reported elsewhere [12,13,24].

We captured female sage-grouse in spring (mid-March through late April) 2008, 2009, 2010, and 2011 and late summer (September) 2009 and 2010. Females were captured using a rocket-net [25] and a CODA netlauncher (CODA Enterprises, Incorporated, Mesa, Arizona, USA) during the spring at and around lek locations. In late summer all females were captured with the CODA netlauncher. We adapted the CODA netlauncher to be a mobile unit, mounting on a truck or all-terrain vehicle (ATV), that made it effective at capturing sage-grouse as sage-grouse can be approached closely (~10–15m) with a vehicle. We fitted VHF radio transmitters (Model A4060; Advanced Telemetry Systems Incorporated, Isanti, Minnesota, USA) to female grouse. Transmitters weighed 22 g (~1.4% of mean female sage-grouse body mass); had a battery life expectancy of 789 days (d); and were equipped with motion-sensors (radio-transmitter pulse rate increased in response to inactivity after 8 hours). We classified sage-grouse as yearlings (first breeding season) or adults (second breeding season or older) based on the shape, condition and coloration of the outermost wing primaries [26,27]. We radio-marked females from 10 leks dispersed throughout the study area to ensure a representative and random sample of the sage-grouse population occupying the study area [28].

We located sage-grouse on the ground using hand-held receivers and 3-element Yagi antennas. We used ground telemetry to monitor radio-marked females through the nesting period (May–June). Nests were confirmed by two consecutive visits that identified the radio-marked grouse using the same shrub or by visually observing the female on a nest with binoculars. We monitored the status of the nests every 2–6 d until the conclusion of the nesting effort. We monitored the nests from a distance of ≥20 m using binoculars or by triangulating to the nest location using radio telemetry in order minimize stress to the female [29]. We retreated in a nonlinear and varying pattern after recording or visiting a nest location to prevent predators from following human scent to the nest.

### Scales and predictor variables

Geographic information system (GIS) data were processed with ArcGIS 10.1 and 10.2 (Environmental Systems Research Institute, Redlands, California, USA), and Geospatial Modeling Environment [30] (http://www.spatialecology.com/gme). We calculated summary statistics for mean sagebrush cover, mean bare ground cover, mean topographic wetness index (TWI) [31], vector roughness measure (VRM) and total conifer for each scale. Summary statistics were calculated using neighborhood statistics that calculated all cell values located within the radius of the target pixel and then assigned the resulting summary statistic to each target cell. We visually checked the accuracy of all spatial predictor variables with ESRI world imagery web map that provides ≤ 1-m resolution satellite and aerial imagery (http://services.arcgisonline.com/ArcGIS/rest/services/World_Imagery/MapServer). World imagery allowed us to visually confirm sagebrush stands, individual conifer and deciduous trees, and CBNG well structures. GIS data layers were created and processed at a 30-m cell resolution unless stated otherwise.

We developed spatial predictor variables at scales known to be important predictors of sage-grouse habitat selection (Table 1). We wanted to focus our assessment on sites contained within energy development, and therefore, we did not assess spatial scales > 1 km^2^ as in other studies of sage-grouse habitat selection [24,32,33]. Energy resources and sage-grouse often co-occur across the sage-grouse range. We assessed the effectiveness of on-site mitigation and therefore limited our scales to those relevant to within energy development. It was not our primary goal to identify the drivers of sage-grouse habitat selection. There have been multiple studies on sage-grouse habitat selection that characterize the influence of landscape scale covariates [11,13,14,24,32–34]. Our objective was to assess the influence of active mitigation on the distribution and abundance of important sage-grouse habitat.

**Table 1:** Spatial variables.

Variable	Range	Description
Well density	pre = 0–8, post = 0–10	Well density as a count of wells within each scale
Roads	pre = 0.00–7.73, post = 0.00–7.85 (km)	Total linear distance of primary roads (regularly used and maintained roads) within each scale
Sage	pre = 4.51–15.79, post = 2.13–16.13 (%)	Mean percent sagebrush (*Artemisia* spp.) cover (Homer et al. 2012) within each scale
Conifers	pre = 0.00–4.59, post = 0.00–7.47 (%)	Percent conifer within scale, assessed as count of 30-m cells containing conifer (Landfire 2010, Version LF_1.2.0)
TWI	pre = 14–2944590, post = 13–3295120	Mean topographic wetness index (TWI; high values = increased soil moisture; Theobald 2007) within each scale
VRM	pre = 0.00–0.05, post = 0.00–0.05	Mean topographic roughness (vector roughness measure [VRM; high values = rougher terrain; Sappington et al. 2007]) within each scale

Spatial variables considered in the development of resource selection function models for greater sage-grouse. The same variables were included in the pre-mitigation (2004 to 2007) and post-mitigation (2008 to 2011) habitat models. Range is presented for each variable summarized at 1 km^2^ for both pre- and post-mitigation landscapes.

Modeling distribution or occurrence of wildlife can be highly sensitive to scale [35]. Therefore, we evaluated predictor variables at two biologically relevant spatial scales: 0.335 km radii (0.35 km^2^) and 0.564 km radii (1 km²). The finer scale (0.335 km radii) was based on the median distance between consecutive year nests of individual sage-grouse in our post-treatment sample (2008–2011). Moreover, earlier research in the Powder River Basin, Wyoming demonstrated that a similar scale (0.35 km radii) was predictive of nest-site selection [24]. The larger scale (0.564 km radii) was based on previous sage-grouse habitat selection research [11,33,34] that found relationships between landscape features and sage-grouse selection at this scale.

Sage-grouse consistently demonstrate strong selection for sagebrush cover across life-stages and regions [11,13,36,37]. Thus, we estimated percent canopy cover that included all species of sagebrush (*Artemisia* spp.; [38]; Table 1).

We included the predictor variable conifer (primarily mountain juniper) as previous research in the PRB demonstrated influences of conifer stands on nest site selection [24]. Conifer data was derived from Landfire products (Landfire 2010, Version LF_1.2.0, http://www.landfire.gov/, assessed 02 August 2013).

Oil and natural gas wells and associated development can affect sage-grouse habitat use [11,13,14,34,39]. We obtained active and plugged and abandoned well data files from the Wyoming Oil and Gas Conservation Commission that included location, type, status, status date, and spud date (initiation of drilling) representing well status at the end of 2011. The unprocessed well data had inaccuracy issues, such as incorrect well location data (e.g., using available imagery we identified well points that were often not accurate to the actual pad location on the ground and there was no spatial pattern to this inconsistency) and, on occasion, a well point would be indicated as having an active status (PG [producing gas]) per the year under consideration but not exist on the ground or a well structure would be visible but lack a well point in the database. We took several steps to rectify the well data to accurately represent the wells that were on the ground during each study year. We first confirmed active wells for each year (2004–2011) by checking the active well data against the plugged and abandoned well data. In addition, we used 1-m National Agriculture Imagery Program (NAIP) and world imagery to inspect the analysis area and validate or correct well locations. NAIP imagery was collected for Wyoming, USA between July–August on a 3-year rotation (2006, 2009, 2012). We then time-stamped wells based on the spud date and batched them into year increments for the entire study period (2004–2011) to depict annual additions or deductions (i.e., wells that were plugged and abandoned during the study). Wells that were drilled (spud date) by May 1 in the sample year were included in that year. Additionally, time-stamped wells were checked for temporal and spatial accuracy using NAIP imagery closest to and succeeding the target year. Further, well accuracy was assessed against as-built plan of development (POD) maps, provided by the BLM-Buffalo Field Office, that were overlaid in GIS. The time-stamped as-built POD maps reflect energy development areas with all associated infrastructure (roads, wells, reservoirs, utility corridors, etc.) immediately after construction was complete and are specific to individual producers.

We classified all wells within the study area as either mitigated or unmitigated. We determined whether a well had been mitigated by identifying the producer as APC and within the APC set separating out wells that were drilled after the 2007 field season, when mitigation was implemented. We confirmed the accuracy of mitigated and unmitigated wells with the as-built POD maps.

Road development in sagebrush habitat results in direct habitat loss and fragmentation [40,41] and has been associated with local extirpations of sage-grouse [21]. Additionally, birds may avoid roads and habitats in close proximity [9] and thus, roads can also result in functional habitat loss. Road traffic and traffic noise has been associated with reduced nest initiation rates, larger lek-to-nest movements [9], declining male lek attendance [23] and possibly lek abandonment [42]. We developed road density estimates across both scales. We visually inspected NAIP imagery and determined that publically available roads layers, such as TIGER/Line 2010 public-domain road layers, were not sufficient to use at the finer scale required for this research. Therefore, to ensure a roads layer that correctly represented the conditions on the ground, we manually digitized primary roads (regularly used and maintained roads) using NAIP imagery at a ~1:3,000 resolution. We were primarily concerned with roads that were used routinely to access wells and other human infrastructure; therefore, our roads layer did not include 4x4 two-tracks. However, because of pronounced differences in surface disturbance and traffic intensity, the interstate (the only paved road in our study area) was not part of our roads layer or corresponding analysis. Roads were time-stamped based on the spud date of the corresponding well site following similar methods as the well layers from 2004 to 2011. Additionally, time-stamped roads were checked for temporal accuracy using POD maps and NAIP imagery closest to and succeeding the target year.

We compiled topographic wetness index [31], and VRM [43] utilizing a 1/3-arc-second National Elevation Dataset (NED; 10-m DEM). TWI is a form of compound topographic index that predicts surface water accumulation on the basis of landscape concavity and hydrology [31] and has been demonstrated to be predictive of nest site selection [24,44]. Terrain ruggedness is known to affect sage-grouse habitat selection at multiple scales [13,33]. Low VRM values indicate flat areas (low slope), moderate values indicate high slope but relatively even terrain (low ruggedness), and high values indicate high slope and broken terrain (high ruggedness) areas.

### Model development

It is important to identify biologically relevant areas that are available for selection by individuals when developing habitat selection models [45]. The selection of a sample of available sites to analyze in a resource selection function (RSF) model assumes that available locations are not known with certainty [28]. Animal movements provide for a biologically meaningful method to define availability. We found that 97% of the post-treatment birds moved ≤ 11 km from the lek of capture to their nest site. Therefore, we defined availability as an 11 km radius around the leks of capture. This distance is consistent with Wyoming statewide sage-grouse movement research [46]. We generated random locations (available units) at a ratio of 5 times the number of used (nest) locations. We employed Wyoming sagebrush products [38] and NAIP imagery to constrain the random locations to available habitats (i.e., sagebrush habitats) by excluding random locations that were inappropriate to be considered available such as exposed rock, open water, conifer stands, tilled agriculture, roads, and hard surface infrastructure pads. For the predictor variables that changed through the study period, such as well and road densities, we batched the random locations with the corresponding nests per year to temporally portray changes in availability.

We modeled sage-grouse habitat use with a use-availability design in an RSF [28] paradigm using logistic regression. We standardized all predictor covariates for the use and available locations for model development. We compared known nest sites to available point locations drawn from within the study area. We selected the best fit term among related variables in the candidate set to develop our multivariate models. We used the complete data set (*i*.*e*., pre- and post-mitigation) to select the best fit spatial scale, and to assess whether a quadratic term was appropriate for certain variables. We calculated selection ratios as the observed count/expected count across 10 quantiles and plotted these ratios across the range of the variable. We assessed the value of including a quadratic term to the variable if inspection of the selection-ratio graph suggested a quadratic relationship, or it made sense biologically (e.g., sagebrush),. We used Akaike Information Criteria (AIC) to compare models with the same descriptive variable to that of a null logistic regression model. The variable was excluded from all subsequent analyses if the variable was not more predictive than the null model. We selected the best fit term for each variable and then calculated the correlation among variables using pairwise Pearson’s correlation coefficients. Highly correlated variables (*r* ≥ |0.60|) were not included in the same model. We used the resulting variables to fit global multivariate models to develop pre- and post-mitigation models which we used to generate spatial predictions.

We evaluated models by partitioning the data using k-fold cross validation with five folds for each model [47]. We iteratively fit global models for each set of training folds and used equal interval bins to group the results into 5 RSF classes. We then calculated the area-adjusted observed number of observations falling into each class. We calculated the Spearman rank correlation between the RSF score and the area-adjusted frequency of validation points for each of the five folds. We also calculated the mean area-adjusted frequency across folds. Models that were good at predicting sage-grouse nesting habitat demonstrated high correlation values among the RSF scores and area-adjusted frequencies.

We developed two global nesting models using the data and methods outlined above. The first model was developed using only pre-mitigation nesting locations to assess the impact of mitigation on abundance and distribution of nesting habitat. This model characterized the distribution and abundance of nesting habitat within the study area prior to mitigation. Model coefficients thus represented estimated relationships in the pre-mitigated landscape. This model characterized sage-grouse response to the landscape (including wells, roads, etc.) prior to mitigation. The second global model was developed using only post-mitigation nest data and hence represented the distribution and abundance of nesting habitat within the study area after mitigation efforts had been implemented. The post-mitigation model coefficient estimates thus represent the relationship among nesting habitat use and the predictor variables post-mitigation and characterize sage-grouse response to landscape features post-mitigation. The pre-mitigation model and the post-mitigation model applied to the same landscape should produce different distribution and abundance of nesting habitat. All model characteristics were kept similar in the development of the two models to allow direct comparison. For example, the same predictor variables at the same scales were used to develop each model and the study site boundaries and available habitat were the same between the two models.

We visualized the model results for key habitat covariates through estimation of the marginal effects for our global models for each time period. We used the margins command in STATA ver. 12.0. We calculated predictive margins across the range of each variable in our datasets. The margins predictions were developed from non-standardized model covariates to facilitate comparison and for ease of interpretation. This allowed us to assess the changes in the probability of use over the range of a covariate.

We applied the pre-mitigation and post-mitigation models to the current landscape within the study area. We overlaid the equal-area-binned models and calculated the change in classification for each pixel by subtracting the bin value of each pixel in the post-mitigation model from the bin value of each pixel in the pre-mitigation model. For example, if a pixel were classified as a 3 in the post-mitigation model and a 1 in the pre-mitigation model, it was assigned a value of 2. Thus, the range of potential pixel values on the overlaid change maps ranged from -3 to 3. We did not predict to cells with well densities higher than those in our models when we applied our models to the landscape. We did not want to predict beyond the extent of our data [48]. Sage-grouse were never observed nesting in areas with well densities > 4 per km^2^. Therefore, these areas were assigned null values in our final maps. We summarized the change in value in proximity of all mitigated and unmitigated well locations.

## Results

Overall, we developed habitat selection models using 488 nest locations. The pre-mitigation data provided by the Wyoming Game and Fish Department ranged from 2004 to 2007 and contained 176 nest locations. The post-mitigation data were collected from 2008 to 2011, included 214 radio-collared females, and contained 312 nest locations. The number of wells in the study area ranged from 499 to 2,581 over the duration of the study. The percentage of mitigated wells within the study site range from 0% in 2007 to 17% in 2011. The average percent sagebrush cover within 1 km^2^ of nests was 11.5% ± 1.6 (Table 1). The maximum well density within 1 km^2^ of an active nest was 5; however, 75% of nests had ≤ 2 wells within 1 km^2^ (Table 1).

All variables included in the global models fit better than a null model. The best fit scale for each variable was generally the larger (i.e., 1 km^2^) scale, with the exception of roads and VRM (Table 2) which were selected at the smaller scale. Quadratic terms were considered for sagebrush and roads and improved model fit (based on AIC) for roads. None of the variables selected for the global models were highly correlated (r > |0.60|).

**Table 2:** Coefficient estimates.

		Pre-mitigation		Post-mitigation	
Variable	Scale	β	SE	β	SE
Sagebrush	1 km^2^	0.30	0.09	0.82	0.08
Well density	1 km^2^	-0.09	0.11	0.18	0.08
Roads	0.35 km^2^	0.51	0.24	0.19	0.16
Roads^2^	0.35 km^2^	-0.22	0.21	-0.13	0.14
Conifers	1 km^2^	-0.62	0.38	-0.37	0.25
TWI	1 km^2^	-1.41	0.33	-1.49	0.30
VRM	0.35 km^2^	-0.35	0.12	-0.05	0.09

Model estimated beta coefficients and associated standard errors for global Resource Selection Function models for greater sage-grouse nesting habitat in the Powder River Basin, Wyoming, U.S.A. Pre-mitigation models were developed using nesting data from 2004–2007. Post-mitigation models were developed using nesting data from 2008–2011. Scale presents the spatial area over which each variable was summarized. TWI: Mean Topographic Wetness Index. VRM: Mean Vector Roughness Measure.

**Fig 2:**
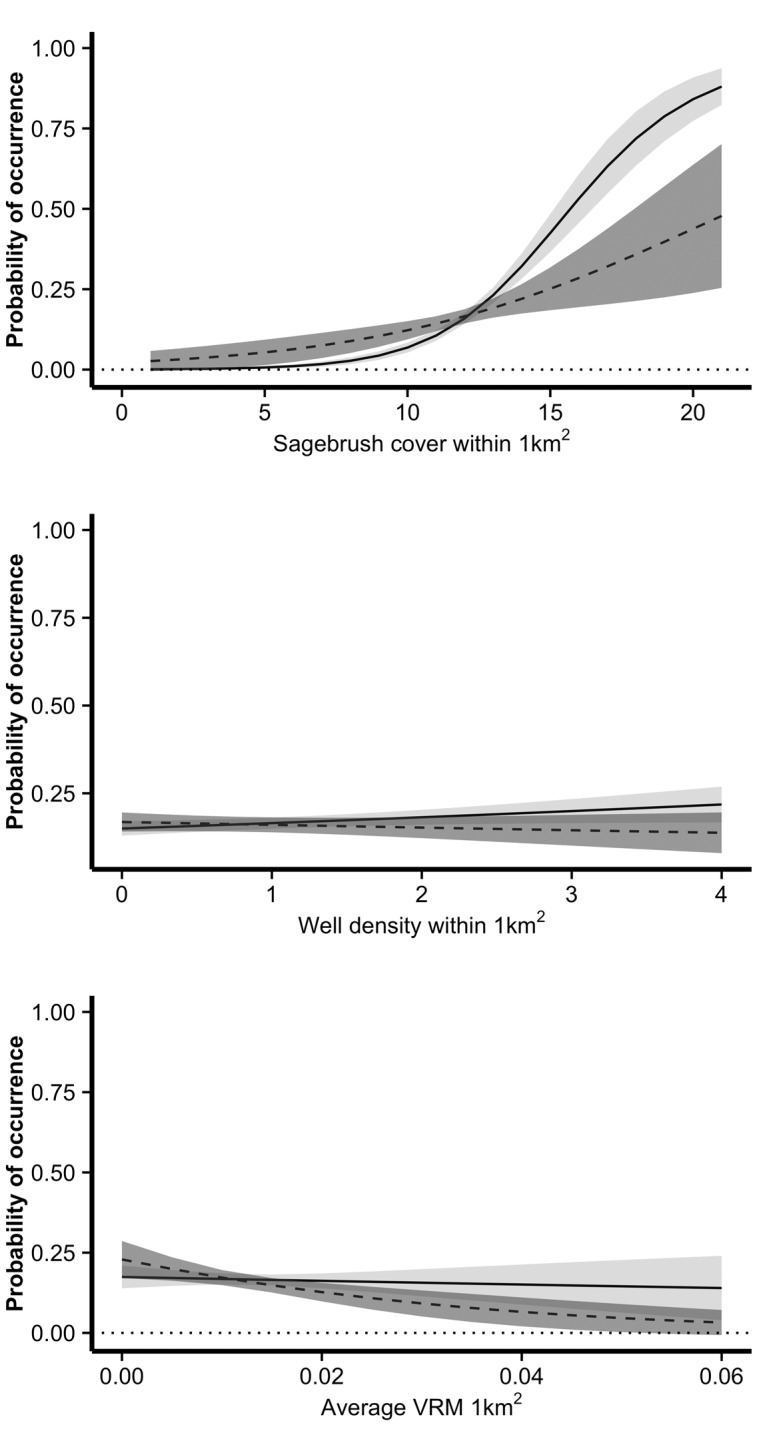
Margins Plots. Margins plot depicting the functional form of greater sage-grouse response to the three variables with different coefficient estimates between the pre- and post-mitigation time periods. Model predictions are based on the global Resource Selection Function models for nesting habitat in the Powder River Basin, Wyoming, U.S.A. Model predictions for the pre-mitigation model (2004–2007) are represented by the dashed lines with darker gray 95% confidence intervals. Model predictions for the post-mitigation model (2008–2011) are represented by the solid lines and lighter gray 95% confidence intervals. VRM: Mean Vector Roughness Measure.

Global models for each timeframe contained 6 covariates representing sagebrush, topographic indices, roads, and well density. The coefficient estimates for the pre- and post-mitigation models were generally similar and, with the exception of well density, in the same direction. However, there were a few covariates for which the standard errors of the coefficient estimates did not overlap between the two time periods (Fig 1). Sagebrush, well density, and VRM coefficient estimates did not overlap. Inspection of the marginal effects plots revealed a generally positive association with sagebrush across the two time periods and a generally flat association with well density across the two time periods (Fig 2). The distribution of these two covariates did not differ at available sites between the two time periods. Spearman rank correlation values between the area-adjusted frequency of validation points and RSF bin across the five folds had an average of 0.82 for pre-mitigation models and 0.96 for post-mitigation models. These average values across the folds suggested that models performed well at predicting nest sites in both the pre- and post-mitigation landscapes and the slightly lower performance of the pre-mitigation models may be due to the smaller sample size.

**Fig 3:**
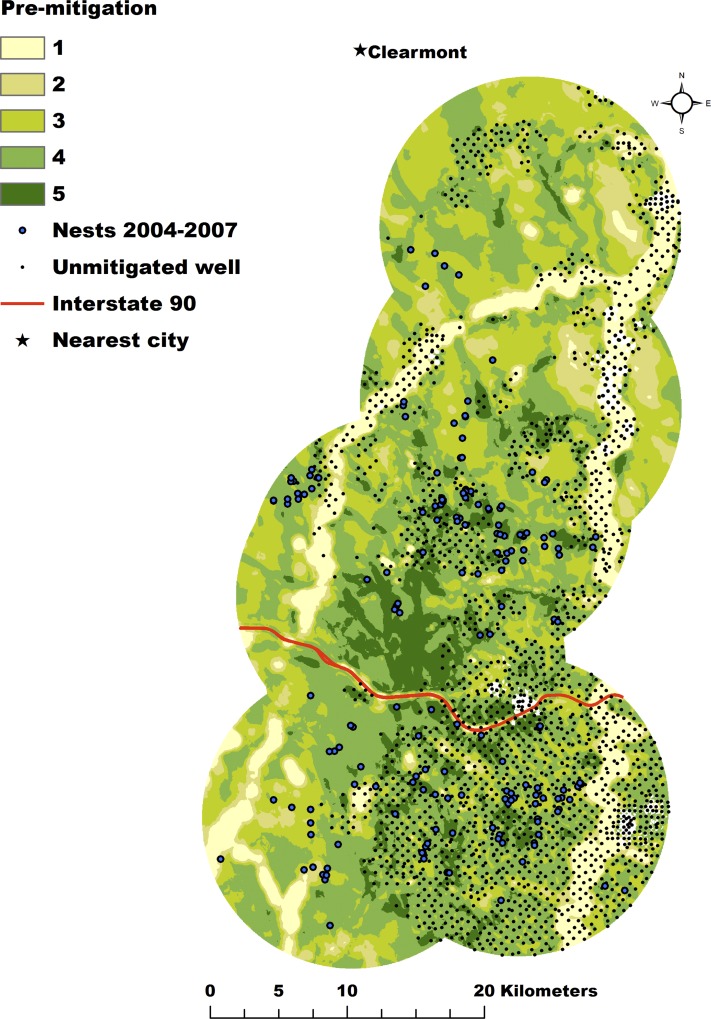
Pre-Mitigation Nesting Habitat. Relative predicted probability surface for the pre-mitigation nesting habitat model divided into 5 equal-area bins for greater sage-grouse in the Powder River Basin, Wyoming, U.S.A. Nest locations used in model development were collected from 2004–2007 and are indicated by the blue circles. The colors range from dark green to yellow to represent the range of relative nesting probability from low to high. Well locations are indicated by black circles.

**Fig 4:**
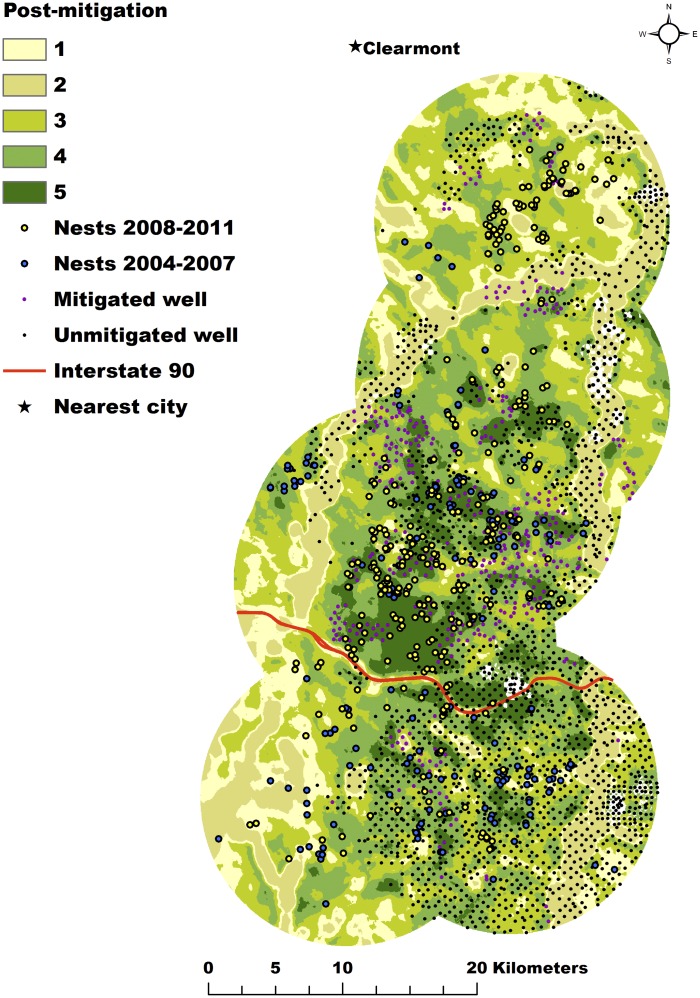
Post-Mitigation Nesting Habitat. Relative predicted probability surface for the post-mitigation nesting habitat model divided into 5 equal-area bins for greater sage-grouse in the Powder River Basin, Wyoming, U.S.A. Nest locations used in model development were collected from 2008–2011 and are indicated by the yellow circles. Nest locations from the pre-mitigation data set (2004–2007) are indicated by the blue circles. The colors range from dark green to yellow to represent the range of relative nesting probability from low to high. Unmitigated well locations are indicated by black circles. Mitigated wells are indicated by purple circles.

**Fig 5:**
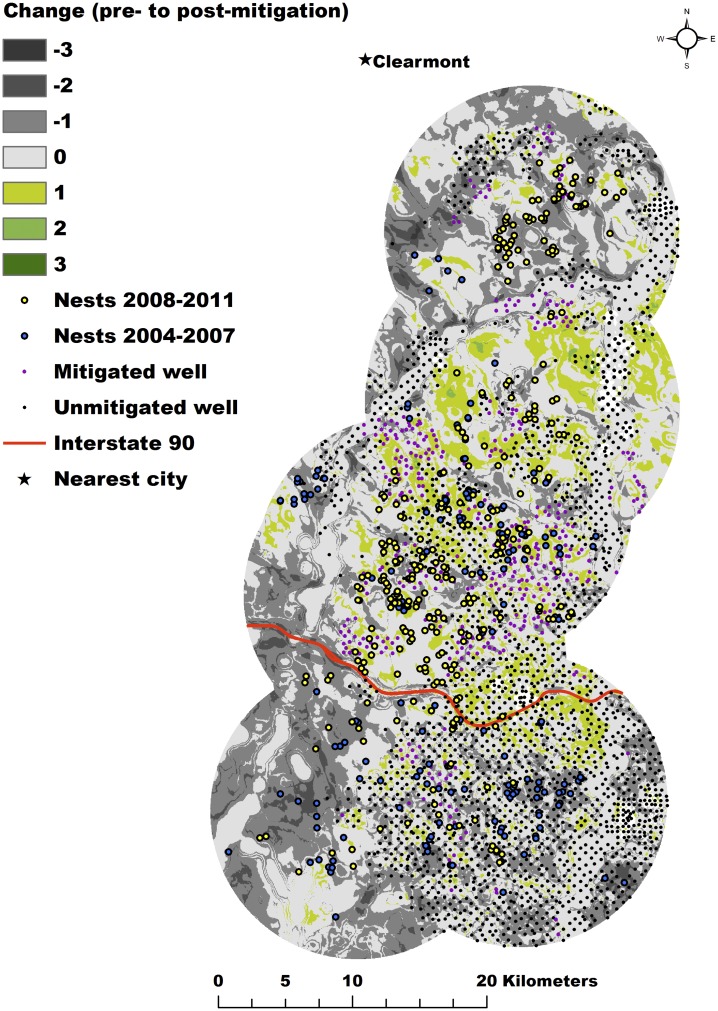
Change in Nesting Habitat. Change in relative predicted probability surface between the pre- and post-mitigation nesting habitat models for greater sage-grouse in the Powder River Basin, Wyoming, U.S.A. Values range from -3 (black) to +3 (dark green) and represent the change in bin classification between the two models from pre-mitigation nesting habitat to post-mitigation nesting habitat. Nest locations used in the pre-mitigation model development (2004–2007) are indicated by the blue circles and the nest locations used in the post-mitigation model development (2008–2011) are indicated by the yellow circles. Unmitigated well locations are indicated by black circles. Mitigated wells are indicated by purple circles.

We projected the results from the pre- (Fig 3) and post-mitigation (Fig 4) models onto the 2011 landscape and binned the predictions into 5 classes, with the higher classes representing more preferred nesting habitat. The overlay of these two maps subtracting the projected pre-mitigation model from the projected post-mitigation model demonstrates generally positive or neutral change throughout the central portion of the study area and generally neutral to negative change throughout the southern and extreme north ends of the study area (Fig 5).

## Discussion/Conclusions

Mitigation efforts seemed to produce measurable improvement in sage-grouse nesting habitats within the boundaries of a development area based on the strength of model coefficients and the observed changes in the distribution of preferred nesting habitats. Sage-grouse response to certain habitat covariates changed between the pre and post time periods. The model coefficients demonstrated less avoidance of wells in the post-mitigation models than pre-mitigation. This suggests that mitigation efforts within the study resulted in less avoidance of wells overall. However, sage-grouse still avoided areas of high density wells. No nests were found in areas with greater than 4 wells per km^2^ and most nests (62.82%) were located in areas with ≤ 1 well per km^2^. Selection for sagebrush also differed between the two time periods with stronger selection (i.e., larger β coefficient) in the post-mitigation period. This indicates that nests were consistently located in areas with higher sage-brush cover. There are several possible explanations for this pattern. First, the quantification of availability can affect coefficient estimates. For example, in areas where sagebrush is less abundant (as estimated by the available locations) birds tend to show stronger selection for sagebrush [33]. The sagebrush data layer used in our models did not differ between the two time periods [38] and if development decreased sagebrush availability over the duration of the study this change would not be represented in the sagebrush layer. However, oil and gas development and associated roads are the primary drivers of short-term habitat change within our study area. Energy development continued throughout the duration of our study and we captured this development in our time-stamped roads and well GIS layers. Therefore, we likely captured the inter-annual variation in habitat distribution through the inclusion of those time-varying covariates. It is possible that the stronger selection in the post-mitigation models is indicative of an on-the-ground change in the availability of sagebrush that was not possible to detect in our sagebrush data layers.

Population density can also lead to competition and competitive exclusion from the highest quality habitats [49]. Sage-grouse in Wyoming (including the Northeast of Wyoming where our study was located) were experiencing a period of population growth as estimated using male lek counts during the pre-mitigation study (2004–2007) [50,51]. There was a turning point in the population trajectory in 2006 that coincided with the implementation of mitigation efforts. Populations were in a steady decline throughout the duration of the post-mitigation study period [50,51]. The increased strength of selection for sagebrush in the post-mitigation models could have been influenced by the lower population densities. Higher densities in the pre-mitigation time frame could have resulted in birds placing nests in suboptimal locations with lower sagebrush cover. This would result in the weaker selection observed in the pre-mitigation models even given similar distribution of available sagebrush habitats.

The extent of a study area—the population of interest—can strongly influence conclusions regarding the functioning of the system. The location of study boundaries influences our understanding of species ecology and can have profound management implications. We intentionally limited our study extent. We wanted to examine sage-grouse habitat selection within an energy development field to assess the localized impacts of mitigation efforts. Our results represent habitat selection behavior *within* energy development fields. Additionally, the roads and well pads within our study extent are developed in some of the best habitat. However, within mitigated development, one of the mitigation efforts focused on avoiding the highest quality sagebrush habitats when siting infrastructure. Energy development can have negative impacts on sage-grouse and birds tend to avoid high density energy developments [9–11,13,52]. The slightly positive coefficient estimates and marginal effects for the well density covariate should be interpreted with caution. Inspection of the marginal effects plots demonstrates wide confidence intervals that suggest the effect should be interpreted as a flat line (Fig 2). It is unlikely that birds are selecting for closer proximity to wells given the weight of evidence provided by previous research. However, the upwards change in the coefficient estimates from the pre-mitigation to the post-mitigation models suggested less avoidance of oil and gas wells in the post-mitigation models.

Not all wells within the study area were the subject of mitigation efforts. Our post-mitigation models contained 17% mitigated wells in 2011. It is possible the limited number of mitigated wells may have masked the potential positive impacts of oil and gas mitigation. This suggests that even limited mitigation can have positive influence on the habitat use of sage-grouse. It is likely that mitigation of the additional 83% of well pads would have lead to even greater changes in the habitat selection behavior of sage-grouse between the pre and post mitigated landscapes.

This study focused on sage-grouse, but multiple other species can be negatively affected by energy development [53–55]. The mitigation approaches assessed here could potentially increase the probability of habitat use within energy development fields by those species also compared to unmitigated energy development. Our study site focused on a CBNG development. However, the mitigation techniques applied are likely applicable to multiple forms of energy development. The development of deep, horizontal wells will likely reduce the overall energy footprint for oil and gas development in the Western U.S. Projected well-pad densities will be reduced (especially when compared to CBNG development) and create less adverse conditions for sage-grouse and other species, particularly when combined with informed on-site mitigation strategies.

Our results suggest that mitigation can affect the habitat selection behavior of sage-grouse within an energy development field. However, habitat is linked with a species fitness components that drive population dynamics and additional work must assess the actual fitness consequences of mitigation. We documented shifts in habitat use, but were unable to address the fitness consequences of these shifts or the source-sink dynamics of the habitat. Future studies that consider the source-sink dynamics of habitat use and potential changes in response to mitigation would be beneficial. It is possible that areas within energy development could be partially mitigated for the use of habitat, but the fitness landscape may not be mitigated, thus potentially resulting in a habitat sink. Previous work has shown that nest and brood [11], and yearling [39] survival can decrease near infrastructure and young birds avoid oil and gas wells when establishing territories. Recent research within our study site suggests that mitigation can have a positive influence on nest success in sage-grouse [56]. The next logical step is to identify the impacts of mitigation on metrics of population performance. Ultimately, understanding the population-level effects of on-site mitigation will be essential to determining its value as a conservation tool.
